# Evaluating effect modification by HIV testing history to understand the mechanisms behind the impact of announcing HIV self-testing availability in a clinic system in Kenya

**DOI:** 10.3389/fpubh.2022.880070

**Published:** 2023-01-06

**Authors:** Elizabeth A. Kelvin, Matthew L. Romo, Gavin George, Joanne E. Mantell, Eva Mwai, Samuel Kinyanjui, Eston N. Nyaga, Jacob O. Odhiambo, Kaymarlin Govender

**Affiliations:** ^1^Department of Epidemiology and Biostatistics, CUNY Graduate School of Public Health and Health Policy, City University of New York, New York, NY, United States; ^2^CUNY Institute for Implementation Science in Population Health, City University of New York, New York, NY, United States; ^3^Health Economics and HIV and AIDS Research Division, University of KwaZulu-Natal, Durban, South Africa; ^4^Division of Social Medicine and Global Health, Lund University, Lund, Sweden; ^5^HIV Center for Clinical and Behavioral Studies, Gender, Sexuality and Health Area, Department of Psychiatry, Columbia University Irving Medical Center, New York, NY, United States; ^6^North Star Alliance, Nairobi, Kenya

**Keywords:** HIV, HIV testing, HIV self-test, truck drivers, sex workers, randomized controlled trial, implementation science, Kenya

## Abstract

**Background:**

In sub-Saharan Africa, truckers and female sex workers (FSWs) have high HIV risk and face challenges accessing HIV testing. Adding HIV self-testing (HIVST) to standard of care (SOC) programs increases testing rates. However, the underlying mechanisms are not fully understood. HIVST may decrease barriers (inconvenient clinic hours, confidentiality concerns) and thus we would expect a greater impact among those not accessing SOC testing (barriers prevented previous testing). As a new biomedical technology, HIVST may also be a cue to action (the novelty of a new product motivates people to try it), in which case we might expect the impact to be similar by testing history.

**Methods:**

We used data from two randomized controlled trials evaluating the announcement of HIVST availability *via* text-message to male truckers (*n* = 2,260) and FSWs (*n* = 2,196) in Kenya. Log binomial regression was used to estimate the risk ratio (RR) for testing ≤ 2 months post-announcement in the intervention vs. SOC overall and by having tested in the previous 12-months (12m-tested); and we assessed interaction between the intervention and 12m-tested. We also estimated risk differences (RD) per 100 and tested additive interaction using linear binomial regression.

**Results:**

We found no evidence that 12m-tested modified the HIVST impact. Among truckers, those in the intervention were 3.1 times more likely to test than the SOC (*p* < 0.001). Although testing was slightly higher among those not 12m-tested (RR = 3.5, *p* = 0.001 vs. RR = 2.7, *p* = 0.020), the interaction was not significant (*p* = 0.683). Among FSWs, results were similar (unstratified RR = 2.6, *p* < 0.001; 12m-tested: RR = 2.7, *p* < 0.001; not 12m-tested: RR = 2.5, *p* < 0.001; interaction *p* = 0.795). We also did not find significant interaction on the additive scale (truckers: unstratified RD = 2.8, *p* < 0.001; 12m-tested RD = 3.8, *p* = 0.037; not 12m-tested RD = 2.5, *p* = 0.003; interaction *p* = 0.496. FSWs: unstratified RD = 9.7, *p* < 0.001; 12m-tested RD = 10.7, *p* < 0.001, not 12m-tested RD = 9.1, *p* < 0.001; interaction *p* = 0.615).

**Conclusion:**

The impact of HIVST was not significantly modified by 12m-tested among truckers and FSWs on the multiplicative or additive scales. Announcing the availability of HIVST likely served primarily as a cue to action and testing clinics might maximize the HIVST benefits by holding periodic HIVST events to maintain the cue to action impact rather than making HIVST continually available.

## Introduction

Several studies have found that offering free oral HIV self-testing (HIVST) increases HIV testing rates in various populations at elevated risk (1–4), including among sex workers (5–7) and truckers (8, 9). However, understanding the mechanisms behind the increased testing rates associated with offering HIVST in these studies is needed to guide the development of HIVST programs to maximize impact. The Health Belief Model (HBM) (10, 11) can contribute to our understanding about the reasons behind the adoption of (or failure to adopt) disease prevention methods and it may be useful in understanding why some people are more likely to accept HIV testing when HIVST is offered. The HBM suggests that six factors influence health behaviors such as the uptake of HIV testing: (1) perceived susceptibility, (2) perceived severity, (3) perceived benefits, (4) perceived barriers, (5) cue to action, and (6) self-efficacy. HIVST might address the perceived barriers that are preventing some people from accessing HIV testing in the standard of care (SOC) programs, such as concerns about confidentiality, inconvenient clinic hours or long wait time, including the time it takes for test administration in the clinic. In addition, offering a novel biomedical technology, like HIVST, could serve as a cue to action, especially when that technology is brand new and not yet available to the public (i.e., if this is someone's only opportunity to try something new or try it for free that may increase their desire to do so).

If HIVST addresses barriers to accessing HIV testing under the SOC, we can expect that making it easily available will have a long-term impact on increasing HIV testing rates. On the other hand, if offering HIVST when it is new with limited availability serves as a strong cue to action for those with an intrinsic motivation to try new things, then we can expect the impact of HIVST to decline over time as it becomes more readily available and the novelty wears off. In one study among truck drivers in Kenya, those offered HIVST had significantly higher testing rates compared to those only offered the SOC at baseline (9), but there was no difference in testing rates over the subsequent 6-month follow-up period when those in the intervention arm were able to pick-up self-test kits at eight participating clinics (12). A majority (79%) of those who chose to self-test at baseline said that the reason for their choice was curiosity to try the new test (9). This may be an indication that the first offer of the HIVST at baseline served as a cue to action for those inclined to try new things and thus increased testing, but after having been introduced to this new technology and having had the opportunity to try it at baseline, it was no longer a novelty and therefore not associated with higher testing rates over follow-up. In addition, some studies have found that when offered a choice among SOC testing, HIVST with provider supervision or HIVST without provider supervision, the majority of those who chose HIVST chose to self-test with provider supervision (1, 5, 8, 9). This may be because some want supervision the first time they self-test to learn the procedure and will later use the self-test without supervision. But it could also be an indication that the impetus to test when offered the HIVST is not because it addressed barriers such as concerns regarding confidentiality or the time required to test with a provider, but instead people wanted to try the new HIV test.

**Figure 1:**
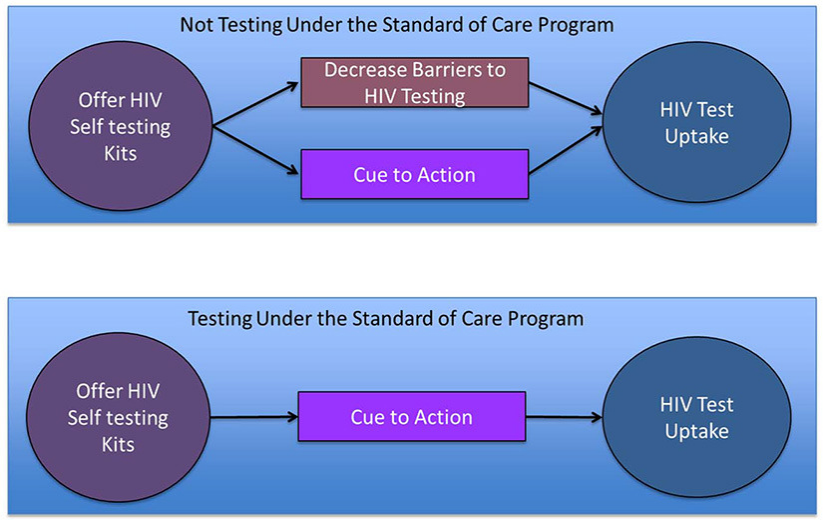
Possible mechanisms by which a new program offering HIV self-testing might increase HIV testing uptake and how they differ by HIV testing history (an indicator for whether the participant faced barriers to HIV testing under the Standard of care that prevented HIV testing in the past year).

To assess whether the impact of HIVST on testing uptake is due to addressing barriers to HIV testing under the SOC versus serving as a cue to action associated with offering a new technology, we might compare heterogeneity of HIVST effect by HIV testing history. If HIVST addresses barriers to HIV testing experienced under the SOC, then we might expect the impact of making HIVST available to be greater among those not previously accessing testing under the SOC. On the other hand, if the HIVST impact is because offering this new technology serves as a cue to action, then we might expect the impact of HIVST to be similar among those who have and those who have not accessed HIV testing under the SOC, or possibly even stronger among those previously accessing testing under the SOC (Figure 1).

Therefore, the aim of the analyses presented here is to assess whether HIV testing history modifies the impact of announcing the availability of HIVST on HIV testing rates among male trucker and female sex worker (FSW) clients of a network of eight roadside clinics in Kenya.

## Methods

For these analyses we used data from two separate randomized controlled trials (RCT), one conducted among male truckers (drivers and assistants) and one among FSWs registered in the electronic health record (EHR) system of the North Star Alliance. The study methods and results have been previously reported (5, 8), but here we give a brief description. The North Star Alliance has established 76 clinics along major transit routes throughout Africa that bring health services to hard-to-reach populations, including sex workers and truckers, with clinic hours that suit the schedules of these target groups. The organization offers a range of primary and secondary healthcare services, including HIV testing and treatment (13). Clients are registered in an EHR system at their first visit to a North Star Alliance clinic, with mobile phone numbers collected if the client is willing, and all subsequent visits are documented in the system. At every client encounter, HIV testing is offered and the test used is the SOC blood-based (finger-prick) rapid provider-administered test. A few times a year a text message reminder about the availability of HIV testing services is sent to registered clients with a valid mobile phone number in the system.

### Sample, eligibility, and consent

The two RCTs were conducted independently at different times but using the same methods. We selected all male truckers (December 13, 2016) and FSWs (February 13, 2017) registered in the EHR who met eligibility criteria: (1) had no indication that they were HIV-positive, (2) resided in Kenya, (3) had a valid mobile phone number listed, (4) had evidence of < 4 HIV tests in the past 12 months in the system [indicating that they were not following the recommendation to test every 3 months (14)], and (5) had not had an HIV test in the past 3 months. The North Star Alliance sent the eligible clients (2,289 male truckers and 2,334 FSWs) a passive consent text message explaining that their data would be used for program evaluation research unless they replied “no” to the text to opt out. Twenty-nine male truckers and 128 FSWs opted out of participating in the study and were removed from the sample for a final sample of 2,260 male truckers and 2,196 female sex workers.

### Intervention

Those who remained in the sample were randomized to one of three study arms: (1) Intervention, which consisted of sending a text message weekly for 3 weeks informing clients that HIVST kits were available at all eight North Star Alliance clinics in Kenya; (2) SOC, which consisted of sending the standard text message one time reminding clients that HIV testing was available at North Star Alliance clinics; (3) Enhanced SOC, in which the SOC text message was sent weekly for 3 weeks.

### Follow-up

We followed study participants for 2 months for the HIV testing outcome. Those in both SOC arms who came to a North Star Alliance clinic were offered only the SOC HIV test, which is offered to all North Star Alliance clinic attendees. Those in the Intervention arm who came to one of the eight North Star Alliance clinics in Kenya were given a brief demonstration of the OraQuick in-Home HIV Test (15) and then offered a choice among (1) the SOC HIV test, (2) the HIVST for use in the clinic with provider supervision, or (3) an HIVST kit to take for home use (provision of pretest counseling in the clinic and posttest counseling over the phone after test use).

### Data collection

For this study, we relied on data from two sources: (1) the North Star Alliance EHR in which HIV testing was documented as per standard procedure, and (2) administrative data collected at the clinics for tracking the number of HIVST kits used to order resupplies and for tracking time since a client took a self-test kit for home use to know when to contact the client if they failed to call for post-test counseling.

The study procedures were approved by the City University of New York Institutional Review Board, the Kenya Medical Research Institute Ethics Committee, and the University of KwaZulu-Natal Biomedical Research Ethics Committee.

### Measures

For these analyses, we combined the SOC and Enhanced SOC groups as previous analysis demonstrated no difference in the HIV testing rates in these two groups (5, 8). Therefore, our exposure of interest was a dichotomous indicator for the intervention (having been sent the HIVST availability text message three times vs. having been sent the SOC message one or three times).

For the HIV testing outcome, we created an indicator for having tested for HIV in the 2 months post intervention initiation (i.e., after sending the first text message) based on evidence in the EHR system or the written clinic records as previous analysis found similar results when the HIV testing outcome included only evidence of HIV testing from the EHR and when we added evidence of testing from the clinic records that was not documented in the EHR at the time of download for analysis (*n* = 5 for truckers and *n* = 38 for FSWs).

The effect modifier of interest for these analyses was HIV testing history, specifically, evidence in the EHR of having tested at least one time in the 12 months prior to randomization.

### Data analysis

The male trucker and female sex worker data were analyzed separately. We described the two samples overall and by 12-month HIV testing history. We assessed the statistical significance of any differences by 12-month HIV testing history using a Fisher's exact test for categorical variables and Wilcoxon rank sum test for numeric variables (i.e., age). We then conducted log binomial regression to compare the proportion HIV tested during the two-month follow-up period among those in the intervention versus the SOC arm overall and stratified on the effect modifier, 12-month HIV testing history. We then added 12-month HIV testing history to the model plus the interaction term for Intervention^*^12-month HIV testing history to evaluate multiplicative interaction. We also assessed interaction on the additive scale, which is perhaps a better assessment of the potential public health impact of an intervention than interaction on the multiplicative scale (16). We calculated risk differences overall and stratified on 12-month testing history and used a linear binomial regression model with the interaction term to test the statistical significance of additive interaction (17). All analyses were conducted using SAS 9.4 (Cary, NC).

## Results

### Description of the male trucker sample

The trucker sample consisted of 2,260 individuals, of whom 750 (33.2%) were randomized to the intervention arm. Median age was 34.0 years and 76.3% were married or cohabitating with a partner. During the study, 51 (2.3%) tested for HIV at a North Star Alliance clinic. Of the 30 truckers in the Intervention arm who tested, 36.7% chose the SOC test, 46.7% chose the HIVST for supervised use in the clinic and 16.7% chose to take an HIVST kit for home use (Table 1).

**Table 1:** Descriptive statistics for the two samples (male truckers and female sex workers) overall and by past 12-month testing.

	**Male truckers (drivers and assistants)**				**Female sex workers**			
		**Indication in health records of HIV test in past 12 months**				**Indication in health records of HIV test in past 12 months**		
**Variable**	**Total**	**Yes**	**No**	**Fisher's exact *p*-value**	**Total**	**Yes**	**No**	**Fisher's exact *p*-value**
Total, *n* (%)	2,260 (100)	607 (26.86)	1,653 (73.14)	NA	2,196 (100)	776 (35.34)	1,420 (64.66)	NA
**Among those who tested in past year, months since last test**								
3–6		379 (62.44)				416 (53.61)		
6–12		228 (37.56)				360 (46.39)		
Study Arm, *n* (%)				0.840				0.638
Intervention	750 (33.19)	199 (32.78)	551 (33.33)		750 (34.15)	270 (34.79)	480 (33.80)	
SOC	1,510 (66.81)	408 (67.22)	1,102 (66.67)		1,446 (65.85)	506 (65.21)	940 (66.20)	
Tested during study, *n* (%)				0.025				0.494
Yes	51 (2.26)	21 (3.46)	30 (1.81)		208 (9.47)	78 (10.05)	130 (9.15)	
No	2,209 (97.74)	586 (96.54)	1,623 (98.19)		1,988 (90.53)	698 (89.95)	1,290 (90.85)	
Age,				< 0.001*				0.007*
Mean (SD)	35.28 (8.66)	34.32 (8.87)	35.62 (8.55)		28.13 (5.94)	28.78 (5.82)	28.78 (5.99)	
Median (Min–Max)	34.0 (18–76)	32 (19–70)	35.0 (18–76)		28.0 (18–61)	27.0 (18–52)	28.0 (18–61)	
Marital status, *n* (%)				< 0.001				0.103
Married/Cohabitating	1,725 (76.33)	398 (65.57)	1,327 (80.28)		176 (8.83)	55 (7.46)	121 (9.63)	
Unmarried (single/divorced/separated)	535 (23.67)	209 (34.43)	326 (19.72)		1,818 (91.17)	682 (92.54)	1,136 (90.37)	
Test selected among those in the intervention who tested, n (%)				0.026				0.009
SOC	11 (36.67)	7 (58.33)	4 (22.22)		48 (40.34)	11 (23.91)	37 (50.68)	
Self-test in clinic	14 (46.67)	2 (16.67)	12 (66.67)		52 (43.70)	24 (52.17)	28 (38.36)	
Self-test at home	5 (16.67)	3 (25.00)	2 (11.11)		19 (15.97)	11 (23.91)	8 (10.96)	

*Wilcoxon rank sum test.

Overall, 26.9% of male truckers had tested for HIV in the past year, of whom 62.4% had tested 3–6 months before the study and 37.6% had tested 6–12 months before the study. The age distribution of truckers who had tested at a North Star clinic in the 12 months before randomization tended to be slightly younger (median age 32.0 vs. 35.0 years, *p* < 0.001), but the proportion married or cohabitating with a partner was higher among those who had not tested in the past 12 months (65.6% of those who had tested vs. 80.3% of those who had not, *p* < 0.001). Those who had tested in the past 12 months were more likely to test in the 2 months following the intervention (3.5% vs. 1.8%, *p* = 0.025) and among those in the intervention arm who tested, those who had tested in the past 12 months were more likely to choose the SOC test over the other two options while those who had not tested in the past 12 months were more likely to choose supervised HIVST over the other two options (tested in past 12 months: SOC test = 58.3%, supervised HIVST = 16.7% and HIVST kit for home use = 25.0%; did not test in past 12 months: SOC test = 22.2% supervised HIVST = 66.7, HIVST kit for home use = 11.1%, *p* = 0.026) (Table 1).

### Description of the female sex worker sample

The FSW sample consisted of 2,196 individuals, of whom 750 (34.2%) were randomized to the intervention arm. Median age was 28.0 years and 8.8% were married or cohabitating with a partner. During the study, 208 (9.5%) tested for HIV at a North Star Alliance clinic. Of the 119 FSWs in the Intervention arm who tested, 40.3% chose the SOC test, 43.7% chose the HIVST for supervised use in the clinic and 16.0% chose to take an HIVST kit for home use (Table 1).

Overall, 35.3% FSWs had tested during the past year, of whom 53.6% had tested 3–6 months before the study and 46.4% had tested 6–12 months before the study. The age distribution of FSWs who had tested at a North Star clinic in the 12 months before randomization tended to be slightly younger (median age: 27.0 vs. 28.0 years, *p* = 0.007). There was no significant difference in the proportion who tested post intervention by testing history (10.1% vs. 9.2%, *p* = 0.494) and among those in the intervention arm who tested, those who had tested in the past 12 months were more likely to choose supervised HIVST over the other two options while those who had not tested in the past 12 months were more likely to choose the SOC test over the other two options (tested in past 12 months: SOC test = 23.9%, supervised HIVST = 52.2% and HIVST kit for home use = 23.9%; did not test in past 12 months: SOC test = 50.7% supervised HIVST = 38.4, HIVST kit for home use = 11.0%, *p* = 0.009) (Table 1).

### Regression results

Table 2 presents the main effect association between the intervention and HIV testing over 2 months post intervention while Table 3 presents the results stratified on HIV testing history (tested in the 12 months before the intervention). Among truckers, those in the intervention had 3.1 times greater probability of testing than those in the SOC (*p* < 0.001) and while this was slightly higher among those not tested in the past 12 months (Risk Ratio [RR] = 3.5, *p* = 0.001 vs. RR=2.7, *p* = 0.010), the interaction was not significant (*p* = 0.683). On the additive scale, the number of truckers who tested in the intervention group was 2.8 per 100 more than in the SOC (*p* < 0.001) overall and this was slightly higher among those who had tested in the past 12 months than those who had not (Risk Difference [RD] = 2.7 per 100 people, *p* = 0.037 vs. RD = 2.5 per 100 people, *p* = 0.003) but interaction on the additive scale was also not statistically significant (*p* = 0.496).

**Table 2:** Impact of announcing HIV self-test availability on HIV testing in the following 2 months for the overall sample.

	**Risk ratio**	**95% confidence interval (CI)**	***P*-value**	**Risk difference per 100**	**95% CI**	***P*-value**
Male truckers	3.12	1.79–5.44	< 0.001	2.81	1.27–4.35	< 0.001
Female sex workers	2.58	1.99–3.34	< 0.001	9.71	6.82–12.61	< 0.001

**Table 3:** Risk ratio and risk difference comparing impact of advertising HIV self-testing vs. SOC stratified on having testing in the past 12 months prior to the intervention and *p*-value for interaction term testing the statistical significance of stratified differences.

	**Tested for HIV in 12 months before study**				**Did not test for HIV in 12 months before study**				**Interaction** ***p*****-value**	
	**Risk ratio (95% CI)**	***P*-value**	**Risk difference per 100 (95% CI)**	***P*-value**	**Risk ratio (95% CI)**	***P*-value**	**Risk difference per 100 (95% CI)**	***P*-value**	**Multiplicative (Risk ratio)**	**Additive (Risk difference)**
**Male truckers**										
HIVST intervention (ref = SOC)	2.73 (1.17–6.38)	0.020	3.82 (0.22–7.43)	0.037	3.46 (1.66 7.21)	0.001	2.45 (0.82–4.08)	0.003	0.683	0.496
**Female sex workers**										
HIVST intervention (ref = SOC)	2.69 (1.76–4.13)	< 0.001	10.71 (5.75–15.67)	< 0.001	2.51 (1.81–3.48)	< 0.001	9.14 (5.59–12.70)	< 0.001	0.795	0.615

Among FSWs we found similar results on the multiplicative scale (unstratified RR = 2.6, *p* < 0.001; tested in past 12 months: RR = 2.7, *p* < 0.001; not tested in past 12 months RR = 2.5 *p* < 0.001; interaction *p* = 0.795) and on the additive scale (unstratified RD = 9.7 per 100 people, *p* < 0.001; among those who tested in past 12 months: RD = 10.7 per 100 people, *p* < 0.001; among those who did not test in past 12 months: RD = 9.1 per 100 people, *p* < 0.001; interaction *p* = 0.615).

To ensure that our results were not overly influenced by the inclusion of those in the intervention with an indication of having tested over follow-up in the written clinic records but not in the EHR data, we reran all analyses only using information in the EHR data. While the associations were slightly weaker, the conclusions remained the same. We also ran the interaction assessment modeling time since last HIV test instead of tested in the past year (>12 months, 12–6.1 months, and 3–6 months) and still found no significant interaction. For truckers, the interaction *p*-value for tested 3–6 months before the study was 0.360 and 6.1–12 months before the study was 0.572 compared to >12 months on the multiplicative scale. On the additive scale the interaction *p*-value for tested 3–6 months before study was 0.959 and for tested 6.1–12 months before the study was 0.291 compared to >12 months. For FSWs, the interaction *p*-value for tested 3–6 months before the study was 0.674 and for tested 6.1–12 months before the study was 0.978 compared to >12 months on the multiplicative scale. On the additive scale the interaction *p*-value for tested 3–6 months before the study was 0.396 and for tested 6.1–12 months before the study was 0.950 compared to >12 months (Data not shown in tables).

## Discussion

For both male truckers and FSWs we found some small variation in the impact of announcing HIV self-testing *via* text message on HIV testing rates over two-months follow-up in both the risk ratio and risk difference by past 12-month testing history, with a slightly greater impact among those who had not tested in the past 12 months on the multiplicative scale and a slightly greater impact among those who had tested in the past 12 months on the additive scale. This difference by scale (multiplicative vs. additive) is to be expected given that HIV testing rates in the SOC were much higher among those who had tested in the past 12 months and background incidence of the outcome impacts the risk difference but not the risk ratio. However, the interaction between history of HIV testing in the past 12 months and the intervention was not statistically significant on either scale, despite the small differences we found in the stratified analysis.

This lack of significant interaction suggests that HIVST is not addressing major barriers to HIV testing associated with the SOC program for truckers or FSWs. Furthermore, most participants in the intervention groups who tested during the study choose HIVST in the clinic with provider supervision (46.7% of truckers and 43.7% of FSWs) while a much smaller proportion (16.7% of truckers and 16.0% of FSWs) chose to take the HIVST kit for home use, which was also found in our previous study among male truckers in Kenya (9) and in a door-to-door testing study in Zambia (1). This suggests that, for the majority who choose self-testing, this choice was not related to a reduction of the barriers related to SOC testing programs, such as lack of time to access the clinic service or concerns about confidentiality. Thus it seems that the most likely mechanism through which making HIVST available increased HIV testing rates in this study and, most likely, in other research studies is through cue to action for those with an intrinsic motivation to try new things. This is further supported by our previous study among truckers, which found that the HIVST intervention was associated with higher testing rates at baseline, right after introducing this new technology, but not over six-month follow-up, by which time the novelty may have worn off (9).

These findings have major implications for the potential impact of making HIVST broadly available. While this might lead to increased testing rates in the short-term, the benefit may diminish over time as HIVST loses its novelty. However, as new HIVST kits are developed and approved, they might be introduced in a way that highlights their novelty (i.e., how they are different from the kits currently available) to extend the cue to action effect of HIVST among those motivated to try new things. Furthermore, with the current roll-out of HIVST kits being primarily in commercial venues for a fee (18), it might be possible to maintain the cue to action impact by holding infrequent events, perhaps once or twice a year, that offer free HIVST kits.

This study does have some limitations to consider. We used EHR data to determine HIV testing both for our outcome and our effect modifier. EHR system data are notoriously messy and our data were no exception, as demonstrated by the fairly high number of participants in the intervention group classified as having tested in clinic records but not in the EHR data (five truckers and 38 FSWs). Furthermore, the EHR data only documents HIV testing through North Star Alliance clinics and it is likely that some participants accessed HIV testing, both before and during the study, at other venues and would have been misclassified as not having tested. Given the randomization of the exposure, the misclassification of HIV testing is likely to be non-differential and therefore would bias the results toward the null, which would make it more difficult to detect effect modification. In addition, we conducted this study among two high-risk groups in Kenya and cannot assume that similar results would be found in different populations who may face different barriers to SOC HIV testing than those in our study. The North Star Alliance makes a concerted effort to facilitate access to healthcare services for truckers and FSWs and therefore their clients may have fewer or different barriers to SOC HIV testing than other groups seeking care in other healthcare systems. HIVST may decrease the barriers to accessing SOC testing faced by other groups even if it does not for those in our studies.

Our findings may be useful in informing the design of HIVST programs for the North Star Alliance clinic system and others that serve similar populations. Instead of adding HIVST to their menu of available services, they might consider holding HIVST events to take advantage of the cue to action effect. Further research is needed to evaluate the mechanisms behind the impact of HIVST in other populations to inform HIV testing program designs that maximize impact.

## Data availability statement

The datasets for this study can be found in the Harvard Dataverse repository with the link: https://dataverse.harvard.edu/dataset.xhtml?persistentId=doi:10.7910/DVN/1DE1A7.

## Ethics statement

The studies involving human participants were reviewed and approved by the study procedures were approved by the City University of New York Institutional Review Board, the Kenya Medical Research Institute Ethics Committee, and the University of KwaZulu-Natal Biomedical Research Ethics Committee. Written informed consent for participation was not required for this study in accordance with the national legislation and the institutional requirements.

## Author contributions

EAK led the conception and design of the study and conducted the data analysis and drafted this manuscript. GG, JEM, EM, and KG were all major contributors to the study design and coordination and participated in data interpretation and manuscript revision. MLR was responsible for data management and study monitoring and participated in data interpretation and manuscript revision. ENN, SK, and JOO were responsible for day-to-day study management and oversight and participated in data interpretation and manuscript revisions. All authors read and approved the final manuscript.
